# A specialist peer mentoring program for university students on the autism spectrum: A pilot study

**DOI:** 10.1371/journal.pone.0180854

**Published:** 2017-07-13

**Authors:** Choo Ting Siew, Trevor G. Mazzucchelli, Rosanna Rooney, Sonya Girdler

**Affiliations:** 1 School of Psychology and Speech Pathology, Curtin University, Perth, Western Australia, Australia; 2 Cooperative Research Centre for Living with Autism Spectrum Disorders (Autism CRC), Long Pocket, Brisbane, Queensland, Australia; 3 School of Occupational Therapy and Social Work, Curtin University, Perth, Western Australia, Australia; Georgetown University Medical Center, UNITED STATES

## Abstract

**Introduction:**

The provision of peer mentoring may improve tertiary education outcomes of students with autism spectrum disorder (ASD). This study evaluated the pilot year of the Curtin Specialist Mentoring Program (CSMP), a specialised peer mentoring program for university students with ASD aimed at improving self-reported well-being, academic success and retention in university studies.

**Methods:**

A single group pre-test, post-test design was employed. Quantitative and qualitative evaluations were undertaken with 10 young adults with ASD to explore the effectiveness and acceptability of the CSMP program. Students completed a battery of questionnaires focused on general anxiety, state communication apprehension, perceived communication competence, and communication apprehension both prior to, and five months after commencing enrolment in the CSMP. Information regarding academic success and retention was also obtained. Interviews with participants provided further insight into their experience of the program.

**Results:**

Students enrolled in the CSMP showed significant improvement in social support and general communication apprehension assessment scores. Interviews revealed key features of the CSMP that may have contributed to these positive outcomes.

**Conclusions:**

The current study provides preliminary evidence that a specialised peer mentoring program can improve the well-being of students with ASD, and highlights the importance of interventions which are individualised, flexible, based on a social model, and target environmental factors such as social support.

## Introduction

Autism spectrum disorder (ASD) is a lifelong neurodevelopmental disability characterised by impairments in social interaction, communication and repetitive or ritualistic behaviours, interests and activities [1, 2]. The prevalence of ASD has increased considerably in recent decades, with the school-aged prevalence of ASD in the United States (US) now approaching 2% [3], primarily as a result in changes in the diagnostic criteria and improved accuracy of diagnosis [4, 5].

Adults living with an ASD are at greater risk of poor social and health outcomes across the lifespan [2, 6–8]. Even for adults without intellectual disability, educational attainment is poor, autonomy in daily life is limited, occupational outcomes are low, and relationships are restricted [7]. Despite the severity of ASD symptomatology declining with age, dependence and social isolation appears to increase [7]. A growing body of research has examined the relative effectiveness of various early interventions in children with ASD [9], however there remains a paucity of research examining the efficacy of interventions aimed at adults [10, 11]. Given the consistent pattern of poor outcomes in adulthood experienced by people living with ASD there is a critical need for programs, which support these people across the lifespan.

The transition from adolescence to young adulthood can be a particularly challenging time for young people living with ASD, as well as for their families [12]. For many, this period of upheaval is compounded by the inadequacies of available support, services, and opportunities [12]. This transition may be particularly problematic for young adults with ASD without an intellectual impairment, with these individuals being three times more likely to have no day time occupation, when compared to adults with ASD who have an intellectual disability [13]. It has been suggested that current post-secondary supports are particularly ineffectual in meeting the unique needs of this subgroup of young adults [13].

It is during this period of transition that many young adults undertake higher education, including those with ASD. In recent years, there has been a substantial increase in the number of individuals with ASD entering university [14, 15]. The reported increases range from two and eight-fold over five years [14, 16]. Preliminary estimates have indicated that the prevalence of university students with ASD is approximately 1%, which is reflective of the rate of ASD in the general population [17].

However despite this increase in enrolment, only a small minority of students with ASD have successfully completed their university education [18]. In Australia, almost a quarter of students with ASD withdrew from higher education before completion [19] and in the United Kingdom students with ASD achieve the lowest percentage of first class or upper second degree classifications across all disability groups [20]. Collectively, these indicators suggest that university students with ASD are not reaching their full academic potential.

Given their cognitive capabilities, the lower retention rate and poorer academic performance of students with ASD is likely related to the non-academic challenges of university life [21]. Difficulties associated with the core characteristics of ASD present a range of challenges including difficulty coping with the lack of structure and predictability of university activities [22], difficulties engaging in academia-related work (e.g. presentations; [22, 23]), and interacting with peers and university staff [23, 24]. Young adults with ASD entering tertiary education also have an increased prevalence of co-occurring psychiatric conditions including social anxiety and depression as well as higher levels of aggression and hostility [17, 25], significantly impacting their ability to cope with the day-to-day challenges of university life.

The idiosyncratic nature of ASD and the high prevalence of co-occurring conditions [17] means that the general supports that are currently available in education settings are often inadequate or inappropriate [19, 26, 27]. Although well-meaning disability support staff offer help, they frequently do not have a clear understanding of the support needs of students with ASD [26]. Adopting a person-centred approach, whereby the student is placed at the centre of the support provision has been reported as the most efficient method of support service [28]. However, it is also important to recognise that as communication difficulties are a core feature of ASD, asking for help is for these students is problematic [27]. Currently there is a need to understand the effectiveness of specialised support programs that are tailored to meet the specific support of university students with ASD [22, 29].

Peer mentoring is emerging as one approach that may be particularly effective in meeting the individualised needs of university students with ASD. The core underlying tenant of peer mentoring programs is the provision of a peer who is able to provide support and guidance on objectives primarily relating to academic or career-related performance or psychosocial objectives [30–32]. Peer-to-peer mentoring has become increasingly common within the university environment, with programs primarily pairing first year undergraduate students with more experienced student peers. Peer mentoring has been shown to improve academic performance [33–35], reduce the stress associated with transitioning to tertiary education [36], increase reports of well-being [36], improve retention [37] and feelings of integration within the university community [38].

While peer mediated programs have been shown to lead to significant improvements in first year students and a number of at-risk student groups, including international students [39], ethnic minority students [40], and students with an intellectual disability [41], few programs have been implemented to assist students with ASD. Peer mediated programs for students with ASD have been implemented at York University in Canada and Sheffield Hallam University in the United Kingdom. While evaluation of the York Asperger Mentorship Program (AMP) is ongoing, preliminary data is promising with a low drop-out rate of student participants from the program [16]. Interviews with participants in the Sheffield Hallam University mentoring program revealed that six out of eight mentees held the program in high regard [42]. Whilst there are indications that specialised programs for students with ASD are able to meet the specific needs of the students, little is known of the effectiveness of peer mentoring for students with ASD attending Australian Universities. This study aimed to evaluate the pilot year of the Curtin Specialist Mentoring Program (CSMP), a program developed for students with ASD attending a tertiary education institute in Western Australia. The primary purpose of this study was to investigate the feasibility of CSMP and its potential for improving well-being, retention and academic performance of students with ASD. It was hypothesised that students participating in the CSMP would report reductions in anxiety, state communication apprehension, general communication apprehension, and increased perceived social support and communication competence. It was also anticipated that participants would report satisfaction with the program.

## Method

### Study design

A one-group pre-test post-test design was employed. This design was deemed to be appropriate given the feasibility nature of the evaluation. Prior to commencement of participation in the CSMP, participating students with ASD completed a questionnaire battery. The questionnaire battery was re-administered 5-months post-enrolment and semi-structured interviews were completed to evaluate the effectiveness of the CSMP.

### Participants

Undergraduate students currently attending Curtin University in Perth Western Australia, who self-reported a DSM-IV diagnosis of autistic disorder or a related condition, and involved as mentees in the CSMP during first semester (24th February to 27th June) of the 2014 Academic Year were recruited. Recruitment was conducted through the Curtin University Counselling and Disability Services (CDS) with mentees of the CSMP program invited to participate in the research study. Of the 12 mentees currently enrolled in the CSMP, 10 (seven males and three females) consented to participate in the research study. The final two mentees did not respond to contact. Participants ranged in age from 17- to 20-years (*M* = 18.0, *SD* = 5.60), and the majority (*n* = 7) were enrolled in the first year of their degree. Further participant demographics are presented in Table 1.

**Table 1:** Participant demographics and course of study.

Participant	Gender	Age	Year of Study	Faculty of enrolment
**1**	Female	19	1	Health Sciences
**2**	Female	18	2	Science
**3**	Male	19	3	Humanities
**4**	Male	19	1	Science and Engineering
**5**	Female	18	1	Humanities
**6**	Male	17	1	Engineering
**7**	Male	18	1	Science
**8**	Male	19	3	Science and Engineering
**9**	Male	17	1	Humanities
**10**	Male	20	1	Humanities

### Intervention

The CSMP is a peer mentoring program based on similar programs provided at Cambridge University in the United Kingdom [22] and York University in Canada (AMP; [16]), as well as strategies in the literature for supporting tertiary students with ASD [43]. The aim of the CSMP is to provide flexible support through one-to-one peer mentoring, targeted at the individual needs of each student. In particular, the program provides students with support in managing the ongoing demands of university life, and encourages the development of skills in self-managing these demands in the future. The peer-mentoring program is centred on the provision of “specialist peer mentors” who are Curtin University postgraduate students, recruited from the School of Psychology and Speech Pathology and the School of Occupational Therapy and Social Work. Mentees are paired with one specialist mentor who provides individualised support based on the needs of the mentees. Topics may include time management, academic performance and communication with teaching staff and peers. CSMP mentee-mentor pairs met weekly for an hour to discuss issues pertinent to the mentee (e.g. managing stress, approaching support staff for help).

Prior to the commencement of the CSMP, mentors underwent specialist training workshops which covered both generic topics (e.g. roles of a mentor, resources available on campus, ways to engage with mentee, confidentiality and boundaries), as well as ASD-specific topics (e.g. discussion about specific mentees based on their assessment profiles, identifying and managing anxiety, social skills training). The individual needs of the mentee were identified and made available to their mentors. Mentors attended a weekly group supervision meeting lead by the two program coordinators, an educational specialist and psychologist experienced in working with young adults with ASD. These supervision meetings aimed to provide specialist mentors with peer-support and a forum through which individual mentee difficulties could be discussed. Further details regarding the training and experience of mentors participating in the program is provided in a separate paper [44].

Mentees and mentors were also encouraged to participate in a weekly Curtin Social Group (CSG), where mentees and their mentors would interact and learn social skills as a group. The activities of the CSG included, meeting weekly for 90 minutes during teaching weeks of the university semester (and included occasional guest speakers), as well as external off campus outings during the university breaks such as tenpin bowling or to the cinema. The CSG provided a safe-environment for the purposes of improving social communication and interaction outcomes.

It was anticipated that peer support through the CSMP would increase feelings of social support, reduce anxiety and communication apprehension and increase communication competence.

### Measures

Evaluation of the program included a battery of questionnaires which measured well-being and communication. Questionnaires were administered face-to-face prior to commencing the CSMP (pre-test), and at the end of Semester 1, 2014, approximately five months later (post-test). Measures of academic success and retention, social validity were also collected at post-test. At this time semi-structured interviews were also undertaken. Outcome measures were selected on the basis that the provision of peer mentoring may improve anxiety, feelings of social support, communication, student satisfaction and retention.

#### Well-being and communication

**Adult Manifest Anxiety Scale-College Version (AMAS-C; [45]).** The AMAS-C is a 49 item questionnaire consisting of yes/no answers. The AMAS -C measures general anxiety in a university student population and yields an overall anxiety score as well as scores on five sub-scales (physiological anxiety, social concern/stress, test anxiety, worry/over sensitivity and lie/validity). To reduce the number of comparisons, only total anxiety scores were analysed and reported. The internal consistency of the AMAS-C is estimated to be .72 to .95, and researchers have reported evidence for construct, convergent and discriminant validity [46]. Higher scores indicate higher levels of anxiety [46].

**Social Provision Scale (SPS; [47]).** The SPS examines the degree to which respondents’ social relationships provide social support. The SPS is a 24 item instrument requiring participants to score each item on a 4 point Likert scale. The SPS covers topics including attachment, social integration, reliable alliance, reassurance of worth, guidance and opportuntiy for nurturance. The SPS has good validity, with a reported internal consistency of .92 [47]. Higher scores indicate greater levels of perceived support [47].

**Situational Communication Apprehension Measure (SCAM; [48]).** The SCAM measures state communication apprehension (defined as anxiety associated with either real or anticipated communication) in a specific context. The SCAM is a 20 item questionnaire requiring participants to rate each item on a 7 point Likert scale. The SCAM typically asks participants to rate the scale items based on how they felt the last time they interacted with someone who had a supervisory role over them. To fit the purpose of CSMP (i.e. promoting self-advocacy skills), the SCAM questionnaire was modified, to instead ask participants to think about the last time they approached someone for help in a university setting. They were then asked to rate how accurately statements described their feelings in that situation. The internal consistency of the SCAM is estimated to be .85 to .90, and the SCAM’s construct and criterion validity have been supported [49]. On the SCAM, item 4 (“I was loose”) and item 8 (“I was ruffled”) were changed to “I was relaxed” and “I was irritated”, respectively, to avoid misinterpretation. Higher scores indicate increasing levels of apprehension [49].

**Self-Perceived Communication Competence Scale (SPCC; [50]).** The SPCC examines an individual’s perceived communication competence in various communication contexts and with different audiences. The SPCC is a 12 item instrument asking questions pertaining to how competent individuals feel they are able to communicate. Participants are asked to rate between 0 and 100, with 0 indicating “completing incompetent” and 100 indicating “competent”. This measure has generated reliability estimates above .85 and substantial predictive validity [51]. Higher scores indicate higher self-perceived communication competence with basic communication contexts and receivers [50].

**Personal Report of Communication Apprehension (PRCA-24; [52]).** The PRCA-24 assesses an individual’s level of communication apprehension. The instrument measures overall apprehension, as well as apprehension in four communication contexts. This measure consists of 24 items, which are rated on a 5 point Likert scale. Reliability estimates of PRCA-24 are generally higher than .90, with high predictive validity [53]. Higher scores indicate higher levels of communication apprehension [52].

Academic success and retention. Information regarding academic grades and study load passed during Semester 1 were also obtained. Participants’ intentions to re-enrol in Semester 2 were surveyed.

#### Social validity

A Student Satisfaction Survey was created based on The Client Satisfaction Questionnaire [54], allowing participants to report how satisfied they were with CSMP. Items were phrased so that they related to the CSMP (e.g. “The program helped me to feel more confident as a university student”; “Overall, I am satisfied with the mentoring program”). Participants answered questions on a five point Likert scale with 1 indicating strong disagreement and 5 indicating strong agreement [54].

As the validity of the standardised questionnaires used in the present study with individuals with ASD has yet to be explored, and in view of the potential difficulties with question interpretation, all questionnaires were completed with the researcher being physically present to answer any questions, if required. This precaution did not turn out to be necessary, and questionnaires were completed in approximately 25 minutes. Good reliability estimates of the various measures were found at both time points (Cronbach’s α ranged between .80 and .96).

Semi-structured interviews were conducted by the primary investigator (CTS) with the participating mentees 5 months after enrollment in the CSMP. The interview guide covered questions pertaining to their experience with the CSMP program, specifically, what factors had been the most helpful, what areas of the program they were not satisified with, and how the CSMP could be improved. In order to facilitate participants’ responses in the semi-structured interview, participants were provided with the interview questions as well as a list of various aspects of university life (e.g. class participation, sense of support) prior to the interview (with approximately 20 minutes preparation and typing time). This was done in order to minimise any potential impact of communication difficulties. During the interview, participants were prompted to clarify or elaborate upon their responses. Some participants appeared to be more comfortable with this interview process and elaborated substantially more than others, accounting for the range in interview times (18 to 43 minutes). Interviews were digitally recorded for later transcription.

### Data analysis

All questionnaire data including any socio-demographic data were de-identified, exported and analysed using SPSS version 21.0 [55]. A generalised linear mixed model (GLMM) was developed to determine whether there was a significant pre-post change on the outcome measures of well-being. The GLMM “robust statistics” option was invoked to accommodate any violations of the normality assumption. A separate GLMM analysis was conducted for each outcome measure in order to optimise the likelihood of convergence. Statistical power was conserved whilst controlling for the inflation of the familywise error rate through the application of alpha correction within groups of conceptually-related outcomes, rather than across all outcomes. Two outcomes were conceptually-related (i.e. state communication apprehension (SCAM) and general communication apprehension (PRCA-24). The GLMMs for these outcomes were therefore evaluated using a Bonferroni-adjusted alpha-level of .025, whilst all remaining outcomes were evaluated at the conventional alpha-level of .05.

Data from the semi-structured interviews was transcribed verbatim and collated with participants’ typed responses. These data were then semantically coded, line-by-line, in units of information as related to the aim of the study. Codes were then collated into potential themes and subthemes and were analysed using inductive thematic analysis as described by Braun and Clarke [56]. A thematic map was developed to understand how each theme and subtheme compared to each other. The themes and subthemes were then refined, and some data were then extracted to illustrate each theme and subtheme.

### Ethical considerations

Written consent to participate was obtained from all participants, including minors (i.e. participants aged 17 years at the commencement of the study) who provided written informed assent. 4.2.8 of the *National Statement on Ethical Conduct in Human Research* [57] states “An ethical review body may approve research to which only the young person consents if it is satisfied that he or she is mature enough to understand and consent, and not vulnerable through immaturity in ways that would warrant additional consent from a parent or guardian” (p. 51). Chapter 4.2 of the National Statement states that minors are able to consent themselves, and do not require parental consent where “young people… are mature enough to understand and consent, and are not vulnerable through immaturity in ways that warrant additional consent from a parent or guardian” (p. 50). This project, including the recruitment and consent procedure, was approved by Curtin University Human Research Ethics Committee (HREC approval number HR 22/2014). Consent from the parents of minors in this study was not required as the minors fit the National Statement description of a mature minor; namely the minors were university students studying at a tertiary institution, able to understand the project and their obligations in providing consent, were not vulnerable and the project was considered low risk. Study procedures and confidentiality of records were maintained in line with the Declaration of Helsinki. Researchers ensured that all participants were aware that they could withdraw from the study at any time without incurring any negative consequences, and Disability Advisors and mentors of participants assisted in reminding participants of this throughout their involvement in the study.

## Results

### Quantitative results

#### Well-being and communication

Mean scores and standard deviations for all measures of well-being and communication are presented in Table 2. Participants in the CSMP showed no significant pre-post change in overall anxiety scores (AMAS-C), state communication apprehension (SCAM) or participants’ perceived communication competence (SPCC). There was a significant pre-post improvement in social support scores (SPS), *F*(1,18) = 4.62, *p* = .045, *d* = .68 (medium to large effect size), indicating that participants felt more supported. A significant pre-post reduction in general communication apprehension was also found (PRCA-24), *F*(1, 18) = 7.66, *p* = .013 (< .025), *d* = .88 (large effect size).

**Table 2:** Pre-test and post-test means and standard deviation on measures of well-being and communication (N = 10).

	Pre-test		Post-test			
	*M*	*SD*	*M*	*SD*	*p* value	Cohen’s *d*
**AMAS-C**	56.70	9.26	54.10	13.49	.084	.58
**SPS^┼^**	72.50	21.67	75.70	11.31	**.045**	.68
**SCAM^┼┼^**	79.10	12.44	80.60	15.49	.854	.06
**SPCC^┼^**	53.66	17.74	59.15	13.11	.187	.43
**PRCA-24^┼┼^**	86.80	10.63	80.80	14.99	**.013**	.88

*Note*. Figures in bold indicate a significant result.

AMAS—C = Adult Manifest Anxiety Scale-College Version. PRACA—24 = Personal Report of Communication Apprehension. SCAM = Situational Communication Apprehension Measure. SPCC = Self-Perceived Communication Competence Scale. SPS = Social Provisions Scale.

^┼^Increasing scores = Improvement.

^┼┼^Decreasing scores = Improvement.

#### Social validity

Overall mean satisfaction score was 4.30 (*SD* = 0.50), out of a total possible score of 5 on the Student Satisfaction Survey. All participants agreed or strongly agreed that they were satisfied with the CSMP, and that the program was appropriate to their needs. In total, nine participants agreed or strongly agreed that they had an increased level of support and eight reported that the program helped them to cope and feel happier about university life.

#### Academic performance

The mean percentage of assessments passed by CSMP participants in Semester 1 was 93.9% (*SD* = 7.76). Participants also achieved distinction (70–79) or high distinction (80+) for 62.9% of units taken, with a failure rate of 2.9%.

#### Retention rates

All study participants re-enrolled in Semester 2 of 2014 Academic Year and within the overall CSMP, with one participant withdrawing from university.

### Qualitative results

**Fig 1:**
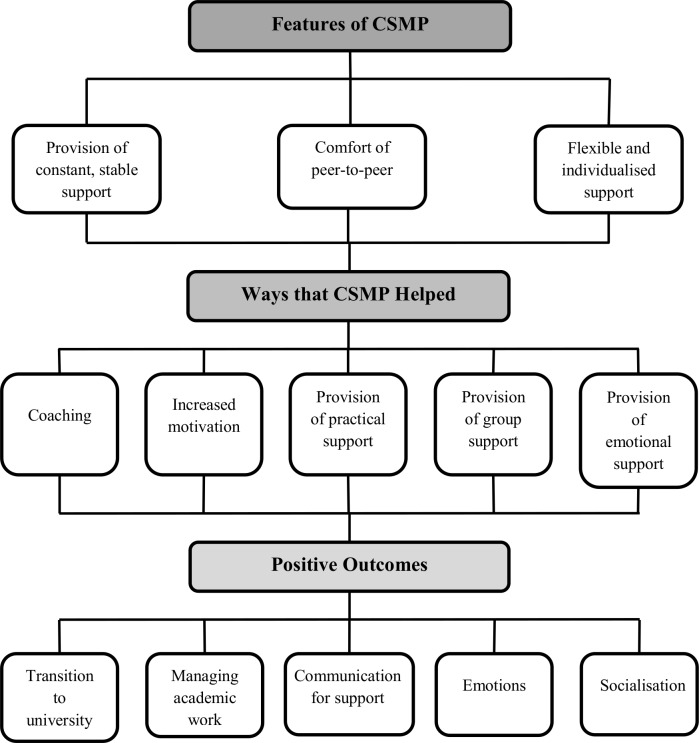
Major themes derived from interviews with mentees related to the features of the Curtin Specialist Mentoring Program (CSMP), how the program helped, and positive outcomes resulting from program.

Based on the analysis of the semi-structured interviews, participants identified (a) specific features of CSMP, which appeared to facilitate the (b) different ways that CSMP was able to help participants in coping with university life, which eventually gave rise to reported (c) positive outcomes (see Fig 1).

#### Features of CSMP

Participants highlighted three positive features of CSMP: (a) provision of constant, stable support, (b) comfort of peer-to-peer support; and (c) flexible and individualised support, as discussed below.

**Provision of constant, stable support.** Nine participants described that they felt confident the program helped them when they encountered difficulties at university. For example, participant 2 reported; “*It’s sort of just*, *in general*, *if I have needed support somewhere*, *I know that I could just text her* [mentor], *and she will be ready to help*.*”*

**Comfort of peer-to-peer support.** Five participants pointed out that having a peer helping them to negotiate the university experience was helpful and that the informal nature of program made the relationship comfortable. Participant 4 stated; “*It is different because it’s more casual…* [it] *isn’t really about building up social skills*, *it just sort of comes along with that by doing activities and talking and stuff like that.”*

**Flexible and individualised support.** Seven participants described how the flexibility of the CSMP was able to meet their individual needs. For example, one mentee mentioned:

*“We don't really have a set thing to go through*, *we just went for an hour each week to discuss what's happened*, *and are there any problems that we can talk about*.*”* (Participant 6).

#### Ways that CSMP helped

Participants identified specific ways through which the CSMP had helped them, as specified below.

**Coaching.** Five participants noted that the program provided opportunities for structured teaching of social skills. For example:

*“We actually went through a bunch of ways you can start a conversation with people*.*”* (Participant 6)

**Increasing motivation.** Seven participants described that the program increased their motivation to “do things”, including university work and pursing social goals. This mainly took the form of the mentors reminding and encouraging them, although some participants also mentioned being self-motivated, so as to not disappoint their mentors. For example:

*“Just pushing me and advising me*. *Because you see*, *having someone there that is not your parents*, *but also an adult*, *an experienced person*, *giving you advice and motivating… you relate to the person a bit differently*.*”* (Participant 7)

**Provision of practical support.** All the participants commented that the program provided one of the following practical forms of support: (a) Collaborative discussions and problem solving, as identified by one of the mentees (Participant 7): *“My financial situation was also a very stressful time for me*, *however my mentor and I came up with strategies and ideas to combat this issue*.*”* (b) Provision of knowledge about how university works. One mentee (Participant 6) stated: “*In class…*. *It is all very directive*, *you can ask questions and stuff*. *And then during lectures… the lecturer is teaching hundreds of students*, *and because he is halfway… talking*, *you can't probably question*. *Adapting to the styles and stuff was pretty helpful talking to* [my mentor].*”* (c) Planning and organising time and academic work. Participant 1 stated: “[My mentor said], *‘Get a calendar*, *write things in it*. *Writing down what you are supposed to be doing for the day and do it*. *Cross it off when you are done*.*’”* (d) Providing company when approaching university staff. For example, Participant 3 commented, *“When you actually talk to someone who works for the university*, *they are helpful and usually very nice*, *but the initial approach is the scary part*. *It’s less so with a mentor there*.*”* (e) Providing knowledge about other support and resources available, allowing participants to obtain support indirectly. One mentee (Participant 10) noted, *“It’s not just like* [support] *from mentoring program or the group*. *Yeah*, *just realising that there is help everywhere*.*”*

**Provision of group support.** Nine participants commented that the group nature of the program facilitated opportunities for socialisation and created a sense of belonging.

*“It’s amazing what it does to have a group of people your own age*, *in the same situation*, *just going through the same stuff that you are*.*… We all tick the same things*, *literally*. *We all used the phrase of speech and everyone will look at us like*, *‘What*?*’*, *and we will be like*, *‘Yep*, *that's totally us*.*’”* (Participant 2)

**Provision of emotional support.** Seven participants highlighted the importance of the emotional support they gained from being able to talk to peers going through the same experiences.

*“It’s not so embarrassing if it is a shared experience*, *if I say or do the wrong thing*.*”* (Participant 3)

### Positive outcomes

Participants described the CSMP as helping them to adjust to university life in several ways.

**Transition to university.** All seven students who were new to Curtin University reported that the program had helped them with transitioning into university life.

“*I think that was the hardest thing* [transition to university]. *So* [the mentoring program] *helped a lot for that*.*”* (Participant 9)

**Managing academic work.** Six participants reported that as a result of the CSMP, they understood their coursework better and were able to complete their assignments more effectively.

“*I understand more easily*.*… Not feeling as anxious as well*, *because I don't know what is going on* [in class].*”* (Participant 10)

**Communicating support needs.** Nine participants pointed out that the program helped them prepare for interactions with university support staff and teaching staff.

“*Planning exactly what to say*.*… So we went over that*, *and I was able to ask the teacher if I can get one* [extension]. *It wasn't that hard*.*”* (Participant 9)

**Emotions.** All participants described the importance of the CSMP helping them to manage negative feelings, as well as instilling more positive emotions. This was broadly categorised into: (a) Managing negative emotions including stress, anxiety and low moods; (b) increased feelings of support; and (c) instilling confidence. The idea that CSMP helped mentee’s manage negative emotions was highlighted by one mentee (Participant 10) who stated, “*University is very stressful for a number of factors*, *namely time constraints*. *The mentoring has helped me find tools to help change this*. *I am less prone than previously to feeling depressed*.*”*, The idea that CSMP increased feelings of support was reflected in the comments of participant 6, “*I'm definitely confident that I will be supported as needed*.*”* Participant 9’s comments reflect the increased confidence gained from participating in CSMP. “*I was initially really nervous about that*. *I was just afraid to say anything because it might be wrong*. *And I guess* [the program] *helped build the confidence a bit*.*”* (Participant 9)

**Socialisation.** Seven participants described the new friendships they had made, both within and outside of the program. One mentee described this friendship experience by stating:

“*So basically for a class*, *just to go and say ‘hi’ to someone*. *Just something as small as that to branch out my acquaintances or network of friends*. *So I met people through friends*, *friends of friends*, *so they become friends*. *So that was very good*.*”* (Participant 4)

### Suggestions to improve or change the program

While overall, participants in the CSMP were very positive about the program, participants did highlight some areas in which the program could be improved. Some participants reported feeling anxious within the program, suggesting that it may have been helpful to commence the CSMP prior to the start of university. Participants also mentioned that the CSG may have benefited from more variation in activities. In particular, while participants found the activities fun, they felt they were repetitive, and at times did not really promote social interactions. Of the 10 participants, only four reported that their experience in CSMP encouraged them to be more independent. While it appeared that the CSMP encouraged mentees to be independent in general, a possible limitation of the peer mentoring model is the potential for over-dependence to develop. Program participants also suggested the CSMP should include more opportunities for socialisation (e.g. informal meet-ups outside of weekly meetings). Although participants were involved in the weekly CSG, it was up to participants to develop these friendships outside of weekly meetings. Thus, the goal of future programs should include promoting independent socialising among mentees. Three of the participants mentioned in the interviews that they had applied the social and organisational skills learnt through the CSMP to settings beyond university, and future programs should focus more on how the skills learnt in a supportive environment can be generalised to other areas of everyday life.

## Discussion

Overall, participants of the CSMP reported high levels of satisfaction with the program. They also reported increased perceived social support and decreased general communication apprehension following participation in the CSMP. Participants in the CSMP also demonstrated a high level of academic performance during the semester, and retention rates were high.

Participants in the CSMP demonstrated a significant increase in social supports between the beginning and end of the semester. While social difficulties present a lifelong challenge for people with ASD [7], the presence of supportive social networks is strongly associated with quality of life among adults with ASD [58]. Previous research has highlighted that university students with ASD perceive themselves as “outsiders” and often feel alienated from campus life [27]. Findings from the present study suggest that environmental factors, such as social support, can be influenced by targeted interventions such as specialised peer mentoring, and also supports the notion that for people with ASD, environmental factors may be more amenable to intervention than impairment factors [58]. It is likely that coordinated and individualised interventions based of social models may be particularly effective in enhancing the supportive networks of young adults with ASD [27].

The most notable change between pre-test and post-test scores was on the measure of general communication apprehension. These findings were consistent with the informally observed changes in the interactions between mentees and mentors within the CSG. Difficulties with communication are a core deficit of ASD and recent research has suggested that the gap between the communication skills of young people with ASD and their peers widens with age [59]. It has been proposed that this is a result of the increasing sophistication of communication patterns during adolescents and early adulthood [59], and the reduction or complete absence of educational and clinical supports available to young people with ASD once they leave secondary education [13]. As communication apprehension may impact a student’s ability to ask for support and guidance, the finding of reduced apprehension in mentees is promising. Individuals with ASD have indicated a need for increased education and social supports [25] and while assistance may be made available, students with ASD may have difficulty asking for such supports. As feelings of support are indicated to play a significant role in retention [60, 61], it is necessary for future iterations of the CSMP to ensure a continued focus on communication. While the findings from the current study are preliminary, they also highlight the potential value of peer mentoring in improving the communication skills of people with ASD into young adulthood.

The results of the present study replicate many of the preliminary findings from other comparable university programs. Similar to mentoring program participants at York University and Sheffield Hallam University, participants in the CSMP were highly satisfied with the program [42]. Within the York University AMP, students reported enjoying the social aspects of the program and the intimate peer-to-peer individual meetings. Specifically, participants cited feelings of social connectedness and support as the aspects of the program they most enjoyed [62] and this was also reflected in comments made by CSMP participants.

Individuals with ASD generally demonstrate a range of different abilities and impairments [24], and CSMP participants likewise reported vast individual differences in the difficulties they faced at university. This may explain why participants recognised the flexibility of the program as a strength, as it enabled the program to be tailored to meet each individual’s needs. This flexibility is a key aspect reflected in programs in other universities. One mentor from the Sheffield Hallam University mentoring program described the support she was offering as “making it up as you go along” [42]. Within the York University AMP, researchers also reported that a large range of topics were covered in individual meetings and that the type of support mentors provided varied according to what mentees were most receptive to [63]. Like those in the present study, participants in these programs also appreciated this flexible, multifaceted support [42].

Previous research has highlighted the need for the development of more individualised approaches to support young adults with ASD [28], as well as services focused on meeting the unique needs of those without intellectual disability [13]. It has been reported that although well-meaning, parents of young adults with ASD may inadvertently restrict the lives of their children and also be perceived as being too controlling [28]. Further, some supports offered by universities can act more as barriers than facilitators to university engagement for students with ASD [27]. Alternate approaches to support, such as peer mentoring, may have other possible benefits including providing age appropriate role models and support in negotiating age-centric environments.

Improvements for future implementations of the CSMP were also suggested. Specifically, participants reported experiencing anxiety within the program. Given that anxiety is commonly associated with ASD [64], future programs may benefit from placing a greater focus on common co-occurring conditions associated with ASD [17]. While specialist mentors were provided with training regarding ASD, further training on common co-occurring difficulties and support strategies may prove beneficial. A separate study that collected feedback from mentors also concluded that mentor training would benefit from more practical ideas on supporting mentees with ASD [44].

It is not surprising that students with ASD identified a need for a broadening of socialisation activities, given that pervasive difficulties in social communication encompass one of the core characteristics of the diagnosis [65]. Adults with ASD have been shown to have fewer peer relationships and recreational activities compared to typically developing adults [66]. Peer relationships contribute to a feeling of integration within tertiary education [38] and are crucial for retention [38]. While traditional peer mentoring programs employed within tertiary education environments may present with an emphasis on academic performance, the results of this study indicate that there is a need for an increased focus on social function within programs targeted specifically at individuals with ASD. The present findings and feedback from participants will inform the refinement of the CSMP training and materials for mentors to guide mentors in how to best support future participants of the program.

Future programs should also aim to promote a generalisation of skills beyond the university setting. Young adults with ASD have significant disadvantages in many areas of adult life including employment, physical and mental health, social relationships and quality of life [67]. They experience difficulty in long-term planning and envisaging their futures [27]. Future iterations of the CSMP could benefit from a greater focus on the transition to life beyond university including linking participants with organisations related to their field of study, thereby providing them with relevant experience and a potential gateway to employment.

A number of limitations must be considered when interpreting the findings of the present study. First, the results must be interpreted with care as the one-group pre-test /post-test study design cannot determine causality or distinguish an intervention effect from confounding Hawthorne effects or other extraneous factors (e.g. communication apprehension could have decreased due to greater comfort in the university). Without a control group, the current study is unable to conclusively provide evidence that the CSMP is the driver for the reported increase in perceived support and decrease in communication apprehension. However, interview data provided some convergent support for these results. In the interviews, all of the participants mentioned that the CSMP helped them in their interactions with other students and staff, with five reporting greater confidence in communicating. Although there was a decreasing trend in college anxiety over the course of the program, the results did not reach significance. While the study detected several significant findings with medium to large effect sizes, the small sample size resulted in limited statistical power. While these preliminary findings provide some validation of the effectiveness of the CSMP, there is a clear need for future research with a more rigorous research design and greater power. Similar to previous research with young adults with ASD, it was challenging to elicit CSMP participants’ views on the support they required [21, 27]. The process of collecting qualitative data from participants was structured to maximise feedback on the program. By obtaining both written responses to interview questions as well as seeking verbal clarification and elaboration, the preferences and abilities of participants in providing feedback was better accommodated. Despite this, only half the participants suggested improvements for the program. Whilst it is possible that the current program is sufficient in meeting their needs, it is also possible that these students have difficulty identifying what is lacking. One participant said, “*You probably shouldn’t be asking me* [what can be improved]. *Because to me*, *I don’t know*, *I wouldn’t have a clue*. *You should probably ask my mum or someone like that*.*”* (Participant 1)

Despite this, it was important to obtain feedback from mentees as they were able to provide insights into the challenges they experienced that were not apparent to others [26]. The present information augments information obtained from mentors [44]. Future research may consider using alternative methodologies such as q-methodology [58]. Confirmation of participants’ ASD diagnosis was not undertaken as part of the present study, and this represents a limitation of the study; although, students involved in the CSMP were allocated to the program through the university disability services prior to enrolment, it was assumed that all students enrolled in the CSMP would present with a diagnosis of ASD. Nevertheless, a diagnostic assessment may have proven beneficial in describing the severity of ASD symptomology present in the current sample.

The current study provided preliminary evidence that a specialised support program can improve the well-being of students with an ASD in a university setting. According to the social model of ASD, disability is constructed as a social difference and not a disorder [23]. This suggests that individuals with ASD are not disabled by their impairments, but more by the dominant societal attitudes of what is considered “normal” (e.g. expected manner of interaction with others). Interventions which meet an individual’s needs while also targeting environmental barriers, such as attitudes, are likely to be the most effective in improving the outcomes of young adults with ASD. While several specialised programs exist and have been implemented at universities, few published evaluative studies are available. The present study serves to extend evaluation outcomes and contributes to the critical examination of the usefulness of such programs.
